# Supplementary figures and images for: Diagnostic value of urinary and serum IgG antibodies in evaluating drug treatment response in strongyloidiasis assessed by fecal examination and digital droplet PCR
Source: PLoS One. 2024 Dec 3;19(12):e0306732. doi: 10.1371/journal.pone.0306732 (PMC11614296; doi:10.1371/journal.pone.0306732)

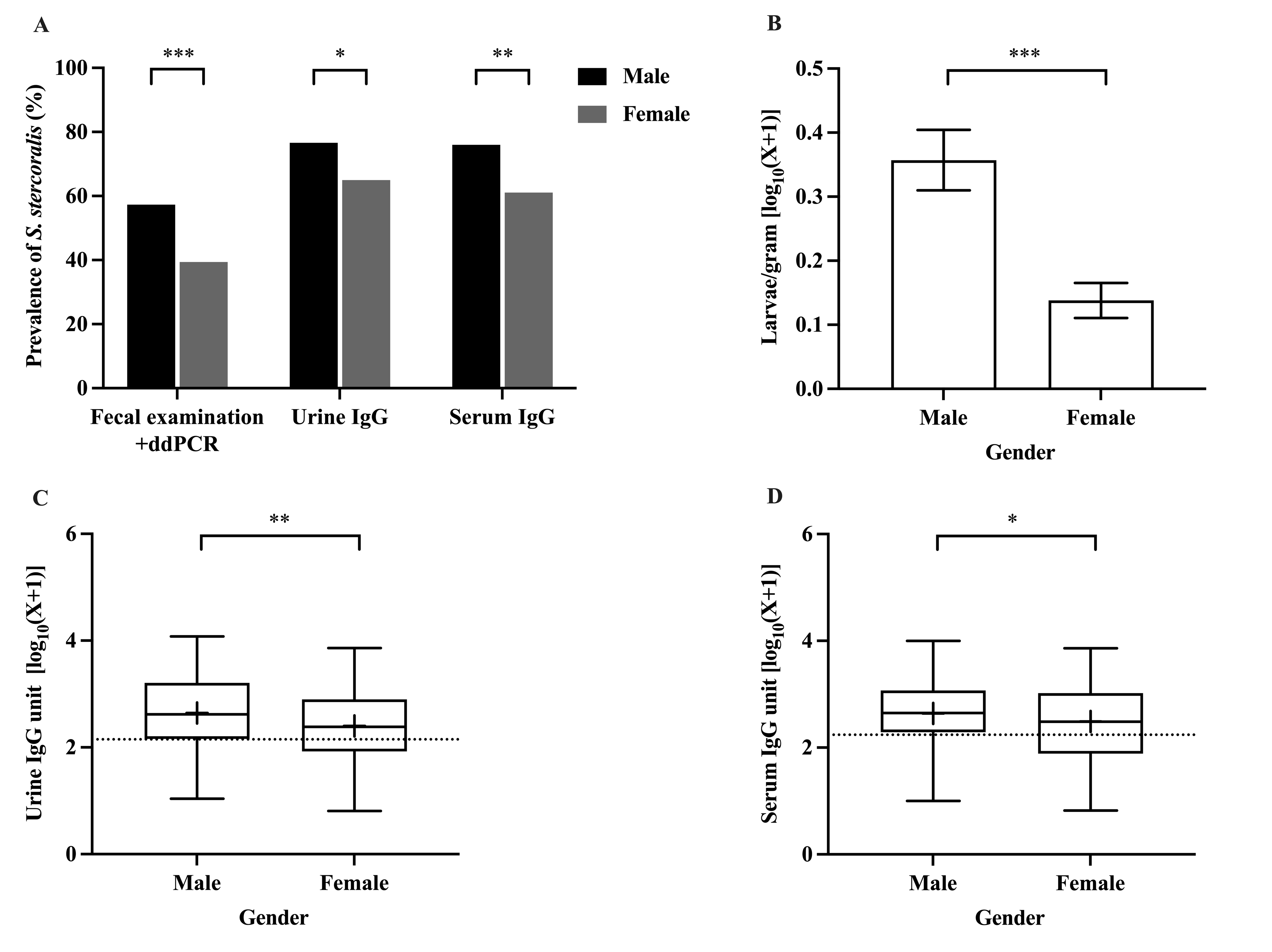

Supplement: S1 Fig — The prevalence of S. stercoralis infection classified by gender by all methods (A). Intensity by fecal examination (B), urine IgG-ELISA (C) and serum IgG-ELISA (D). The graphs with box whisker plots represented min-max with median and + symbol represented mean value. (TIF) [file pone.0306732.s001.tif]

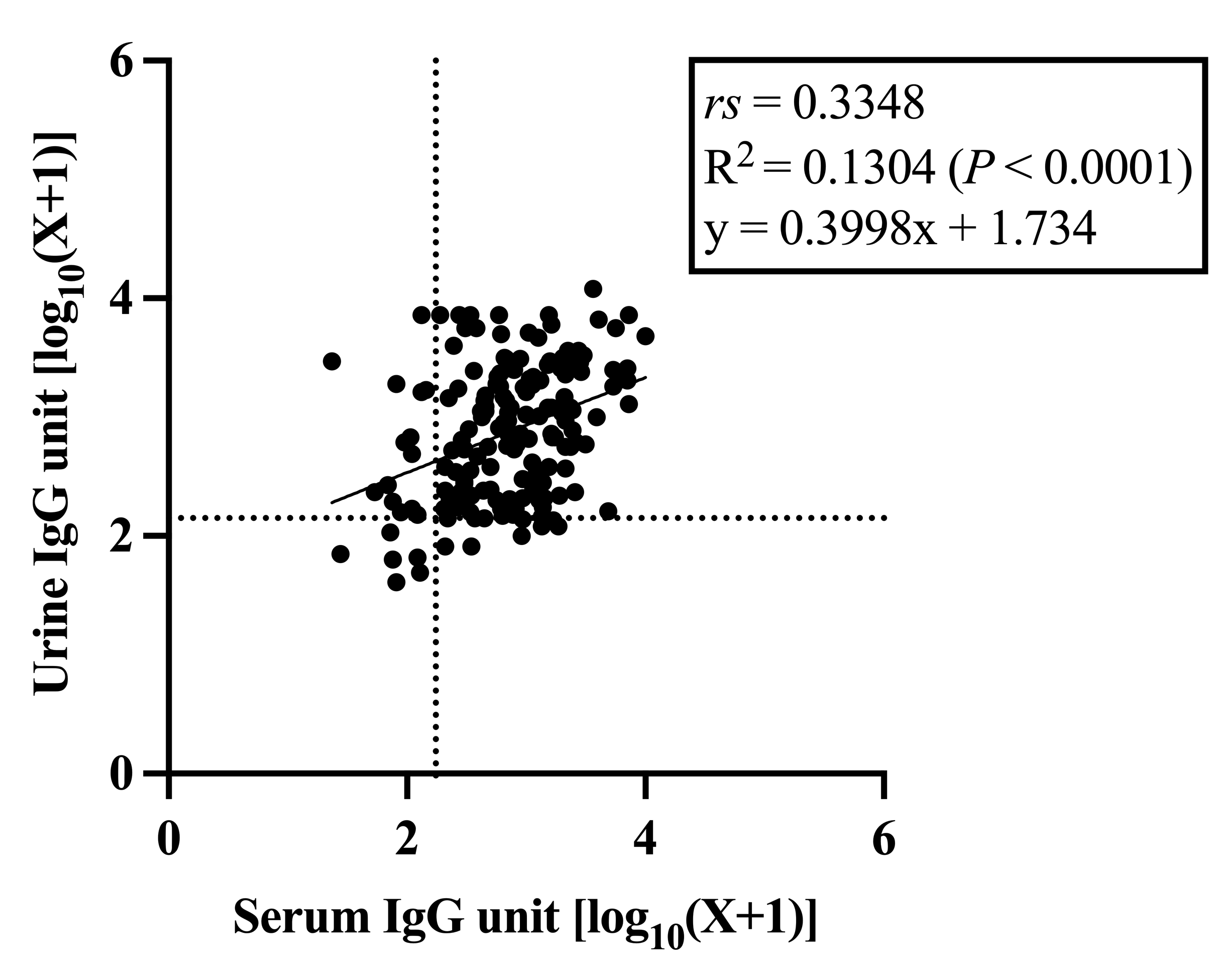

Supplement: S2 Fig — Data shown are observed values and the solid line was calculated from the regression equation (Y = ax+b, Y = log IgG, a = slope, x = log IgG, b = Y-intercept). Dotted lines (vertical and horizontal) represent the cutoff values. (TIF) [file pone.0306732.s002.tif]

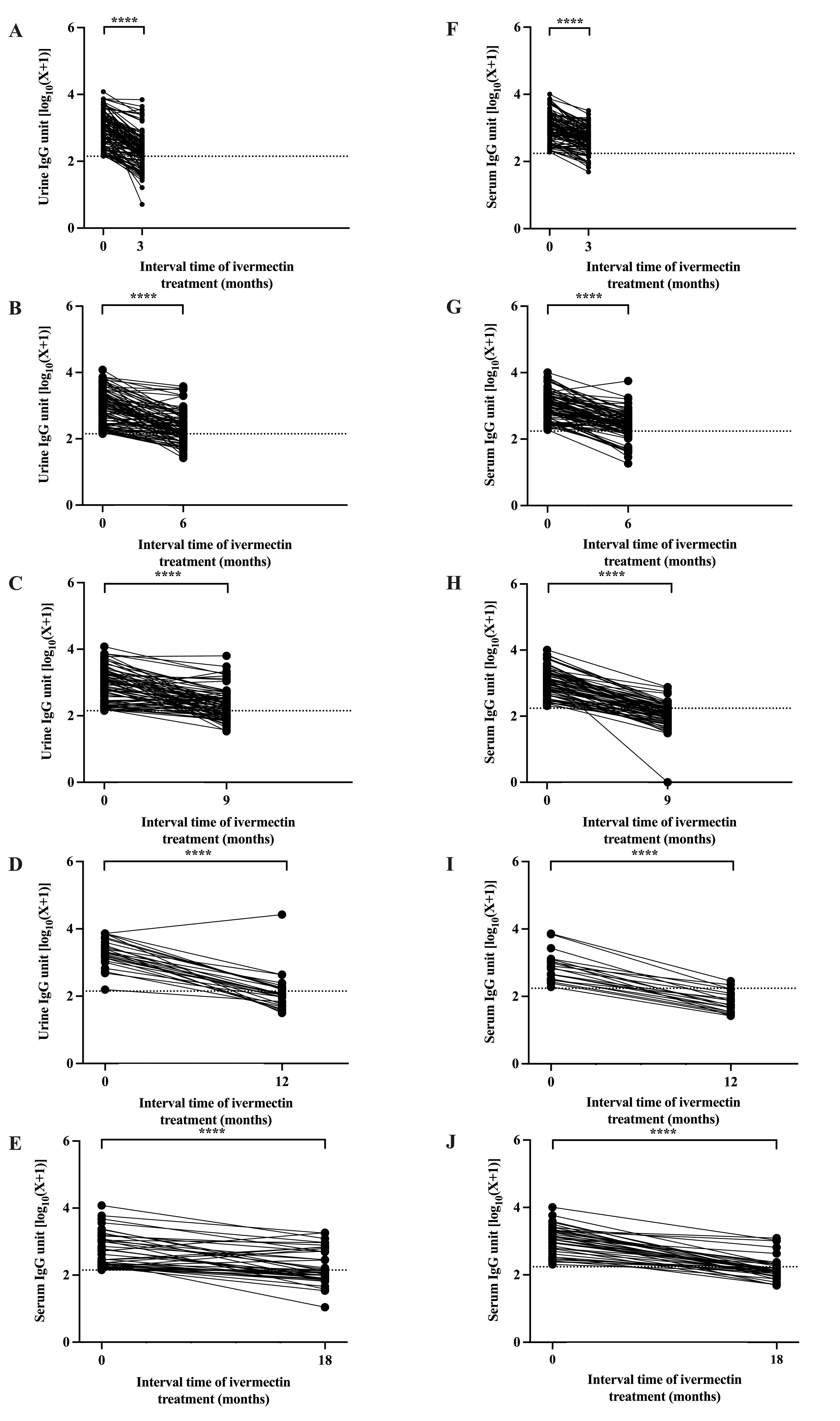

Supplement: S3 Fig — Strongyloides-specific IgG in urine (A-E) and in serum (F-J). Significant different between pair samples by Wilcoxon matched-pairs test, **** p value < 0.0001. (TIF) [file pone.0306732.s003.tif]

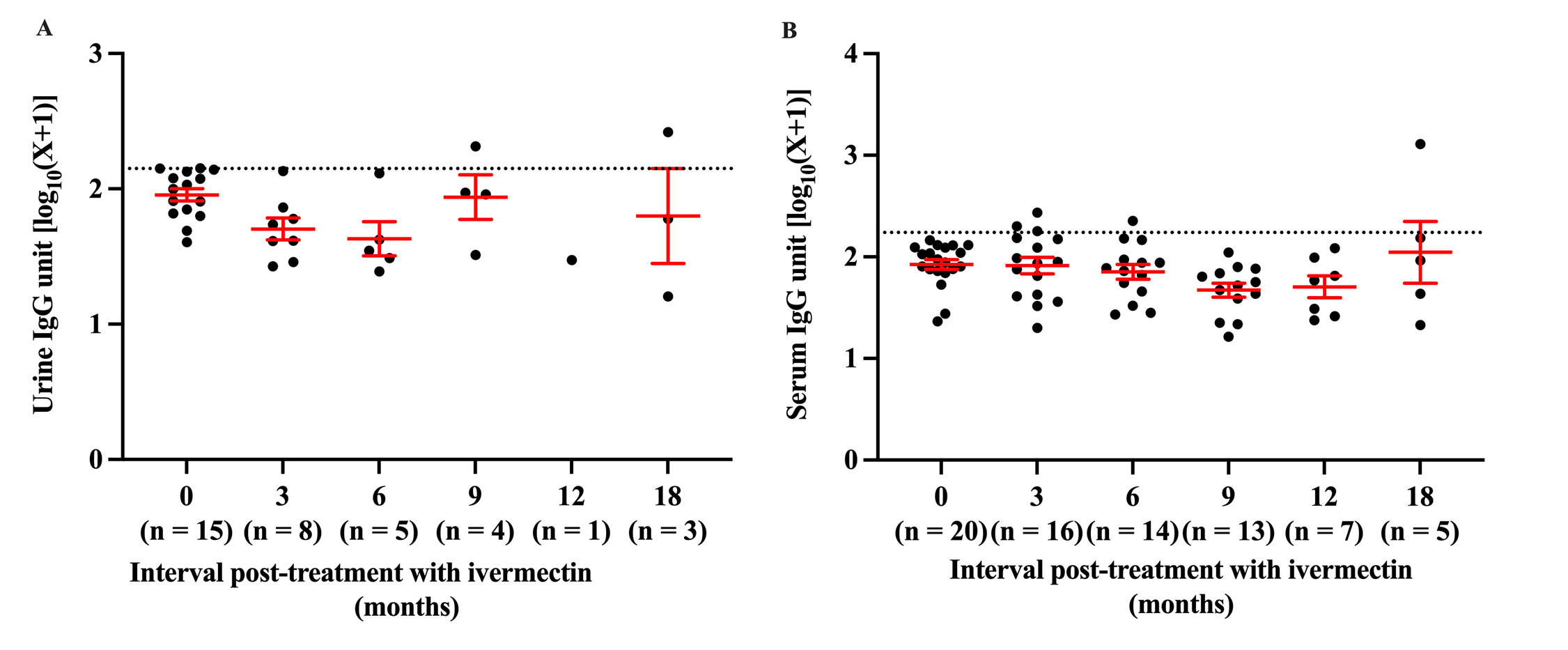

Supplement: S4 Fig — Strongyloides-specific IgG antibody units in urine (A) and serum (B). Data points were individual values and error bar represents mean ± SE of IgG antibody units/ml (log10[X+1]). (TIF) [file pone.0306732.s004.tif]

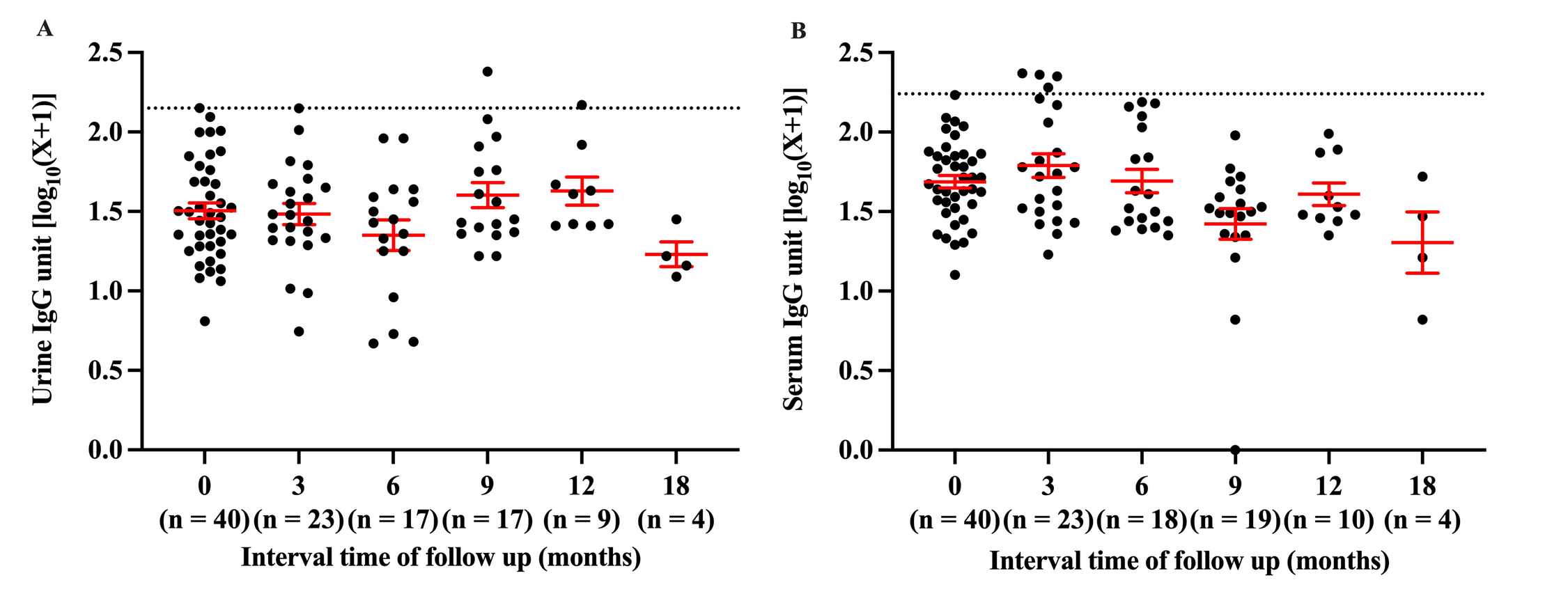

Supplement: S5 Fig — Strongyloides-specific IgG in urine (A) and serum (B), respectively. Error bar represents mean ± SE of IgG antibody units/ml (log10[X+1]). (TIF) [file pone.0306732.s005.tif]
